# Internet use and health in higher education students: a scoping review

**DOI:** 10.1093/heapro/daab007

**Published:** 2021-03-19

**Authors:** Hanna Rouvinen, Krista Jokiniemi, Marjorita Sormunen, Hannele Turunen

**Affiliations:** 1 Department of Nursing Science, Faculty of Health Sciences, University of Eastern Finland, Yliopistonranta 1C, PO Box 1627, Kuopio FI-70211, Finland; 2 Institute of Public Health and Clinical Nutrition, Faculty of Health Sciences, University of Eastern Finland, Yliopistonranta 1C, PO Box 1627, Kuopio FI-70211, Finland

**Keywords:** Internet, health, student, higher education

## Abstract

The amount of time spent online has increased over the last decade among higher education students. Students engage in online activities related to studies, work, leisure, entertainment and electronic services (e-services) use. The Internet is also used for health-related matters. The increase in the use of the Internet has influenced students’ health, especially mental and physical health and well-being. This scoping review scrutinizes the literature between 2015 and 2020 (*N* = 55) on the association between Internet use and health in higher education students. A methodological framework, outlined by Arksey and O'Malley, was applied to conduct this review. Systematic searches were carried out in the CINAHL, PubMed and Scopus databases and in the available grey literature. For the data, a thematic analysis by Braun and Clarke was utilized. Two major themes of ‘Health-promoting Internet use’ and ‘Health-threatening Internet use’ emerged and are described in this review.

**LAY SUMMARY**

Internet use for higher education students is a way of life, and for some, it is even a problem. Previous research has identified Internet use effects on health, especially on mental and physical health. Our research indicated that Internet use has positive effects (promoting) or negative effects (threatening) on health among students. We believe that the results of this review can be utilized in promoting higher education students’ health and well-being.

## INTRODUCTION

The Internet, the global system of networks, is characterized as one of the most significant information-finding and sharing forums that higher education (HE) students use daily (Geyer *et al.*, 2017). Students exhibit a high level of competency in Internet use with digital technologies, such as smartphones or tablets (Essel *et al.*, 2018; Lepp *et al.*, 2019). According to previous studies, HE students’ daily Internet use varies from fewer than four hours to over eight hours, with the average being four to five hours (Al-Gamal *et al.*, 2015; Qader *et al.*, 2015; Sumaiyah Jamaludin *et al*., 2018). Students engage in online activities related to studies and work, leisure and entertainment and the use of electronic services (e-services) (Geyer *et al*., 2017; Mou *et al.*, 2017; Chern and Huang, 2018). Additionally, health-related Internet use is common. Students use online health information to address or solve a health problem and communicate about health issues online (Mou *et al.*, 2017; Yang et al., 2017). The use of health services provided online—as well as web-based health interventions and treatments—is increasing (Merchant *et al.*, 2017; Mou *et al.*, 2017).

Against the positive sides of HE students’ online activities, Internet use has become a problem for growing number of students, ascending to pathological or addictive Internet use (Young and de Abreu, 2011; Li *et al.*, 2015; Kumar and Mondal, 2018). This problematic Internet use is described by numerous terms, for instance, ‘excessive Internet use’, ‘psychopathological Internet use’, ‘problematic Internet use’, ‘Internet dependence’, ‘iDisorder’ and ‘compulsive computer use’ (Nath *et al.*, 2016; Li *et al.*, 2018), meaning a negative influence on various interpersonal, social, psychological and physical health domains of students’ life (Maurya *et al.*, 2018). Students with problematic Internet use exhibit obesity and sleep disorders (Li *et al.*, 2016), comorbid mood and anxiety disorders (Kuss and Lopez-Fernandez, 2016) and behavioral problems, such as sedentary lifestyles and lower levels of physical activity (Penglee *et al.*, 2019). However, effective professional treatments exist to address these issues, for example, new clinical centers have been established to treat Internet-use-related problems (Kuss and Lopez-Fernandez, 2016).

During the HE years, students undergo a transition to adulthood. They are in a developmental stage when autonomy from their parents is increased (moving away from the family home) and changes in financial status are experienced. Students are known to experience demanding studies, pressure to graduate and make career choices (Aceijas *et al.*, 2017; Auerbach *et al.*, 2018). Additionally, HE students’ abilities, self-regulation and overall control are developing, and therefore, physical and mental developments are still evolving (Shao *et al.*, 2018). Hence, the HE era is associated with taking part in risky health behaviors, such as substance use, risky sexual behavior, not getting enough sleep, not eating healthily and being sedentary more than recommended **(**Evans-Polce *et al.*, 2016; Mou *et al.*, 2017; Mnich *et al.*, 2019; Vainshelboim *et al.*, 2019). Above all, contemporary HE students consider themselves healthy, even though they suffer from different health symptoms, illnesses or injuries. Among students, the prevalence of various diseases has continued to exist at a somewhat unchanged level, whereas diagnoses of depression and anxiety syndrome have almost tripled since the year 2000 (Kunttu *et al.*, 2017). Anxiety, together with stress, continues to be the leading health concern among the HE student population (Calamidas and Crowell, 2018). A considerable amount of research to date has studied HE students’ health and influential factors. However, the literature is not as co-directional regarding the implications of Internet use effects on health, as the evidence is still emerging.

This scoping review aims to present a wide-ranging view of the current literature between 2015 and 2020 on the association of Internet use and health in HE students. The phenomenon is approached with a holistic perspective, meaning that Internet use is viewed without categorizing the use to problematic use or to specific online activities. Health is approached from a comprehensive viewpoint, considering physical, mental, social, spiritual and emotional dimensions (Eberst, 1984). The consistent conceptualisations vary in the literature on how Internet use is described and how health is approached, despite which the research is growing. In addition, the assessment and classification of the association between Internet use and health is multidimensional. It is expected that the results of this review may help identify gaps and indications for future research on the topic. In addition, this review’s intention is to summarize findings in an accessible way to inform evidence-informed policy and practice at HE levels. As far as we know, no other scoping review with this topic, on this population has yet been published. However, reviews on Internet addiction and problematic Internet use effects on health exist (Kuss and Lopez-Fernandez, 2016; Hinojo-Lucena, *et al.*, 2019).

## METHODS AND ANALYSIS

A scoping review was performed to identify and explore literature on the association of Internet use and health among HE students. The review was carried out using a framework defined by Arksey and O’Malley (Arksey and O'Malley, 2005) for scoping reviews. Consistent with the methodology, the review was executed in five stages as follows: (i and ii) research question and the relevant articles identification; (iii) article selection; (iv) data charting; and (v) results collating and summarizing, as well as reporting. The sixth stage, which encompasses an optional consultation, was left out of the process.

### Research question identification

The objectives of this review were to map the accessible literature on the association of Internet use and health among HE students and to describe the key findings and identify emerging themes. The broad question addressed for the review was: ‘what is known from the existing literature about the associations between HE students’ Internet use and health?’ The certain inclusion and exclusion criteria were set according to the Population-Concept-Context (PCC) framework to define the research question (Joanna Briggs Institute, 2019) (Supplementary File S1).

### Identification of relevant articles

**Fig. 1:**
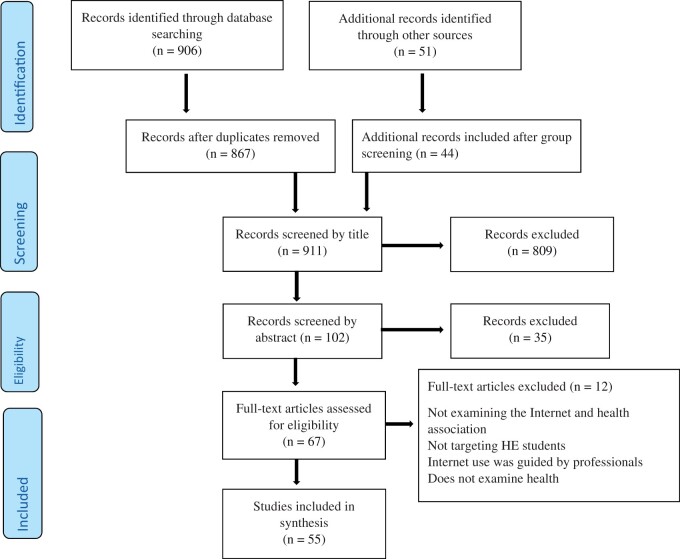
PRISMA flow diagram of the search and evidence selection process (Source: Moher *et al.*, 2009).

Key concepts underpinning the research area were identified and clarified to align with the research question. In doing this, the key search terms were developed. An academic librarian confirmed the search strategy. Search terms were ‘HE student (university, college, tertiary, polytechnic)’; ‘Internet (net, web, online activities, social media, smart/mobile device) use’; and ‘health’. An extensive search was conducted in the electronic databases of CINAHL, PubMed and Scopus. Additionally, a search of the relevant grey literature was carried out to include the World Health Organization (WHO) Library database (WHOLIS), Google and Google Scholar search engines and dissertation databases. Additionally, targeted websites of relevant national organizations, such as The Finnish Student Health Service, The Research Foundation for Studies and Education, The Finnish Society of Media Education and The Family Federation of Finland, were searched. Experts from these national organizations were consulted. Furthermore, manual searches of the reference lists of all selected articles were conducted. When articles were unavailable, authors were contacted. The time limit for the searches was 6 years, 2015–2020. The language was limited to English, Finnish or Swedish, with articles addressing evidence globally. Search results were exported to ProQuest RefWorks to be further reviewed (ProQuest L. L. C, 2020). The selection process is reported as recommended by the PRISMA statement (Moher *et al.*, 2009), which is also recommended for scoping reviews in the PRISMA Extension for Scoping Reviews (PRISMA-ScR) (Tricco *et al.*, 2018) (Figure 1).

### Selection of articles according to the established criteria

Two reviewers (H.R.) and (K.J.) screened all articles independently for eligibility and to establish interrater reliability. This was performed with a developed screening matrix and used with Microsoft Excel (Microsoft Corporation, 2020). The Cohen’s kappa coefficient with a 95% confidence interval was counted to determine interrater agreement for the consistency of screening (Stemler, 2004). It was calculated using the number of includes and excludes during the three-round review process. Kappa results indicated substantial level of agreement (0.79, 0.64, 0.62) (McHugh, 2012). Disagreements on the eligibility of the article for inclusion were discussed and resolved through consensus. One reviewer (H.R.) conducted the grey literature search using the same criteria and phases of article selection. Furthermore, the selection of studies and literature was executed in consultation with the review team.

### Charting the data

A ‘descriptive analytical method’, as described by the review methodology, was used to extract information on the included articles. This technique included sifting, charting and sorting material for synthesis and for data interpretation (Arksey and O'Malley, 2005). Articles were categorized by author information, study/article objective, study/article design and sample, outcome measures and main findings (Supplementary File S2).

### Collating and summarizing the results

As typical with scoping reviews, a descriptive summary and a thematic analysis of the included articles were conducted (Arksey and O'Malley, 2005). The analysis was performed in stages, thusly: the article data familiarization; generating and searching for codes and themes; reviewing and defining the themes, and writing the final report (Braun and Clarke, 2006). An example of the analysis process is presented in Table 1.

**Table 1:** An example of thematic analysis process with their associated codes

Data	Code	Sub-themes	Theme	Main theme
The same technologies also offer several opportunities for the enhancement of mental health and the treatment of mental illness (Lattie *et al.*, 2019).	Internet technologies offer opportunities for the enhancement of mental health	Promoting factors for mental health and well-being	Promoting and threatening factors for mental health and well-being	Internet use and health among higher education students: health promoting and health-threatening factors
Internet technologies offer opportunities to treat mental illness				
Excessive Internet usage leads to anxiety, depression and adverse mental health (Lebni *et al.*, 2020)	Excessive Internet use leads to anxiety Excessive Internet use leads to depression Excessive Internet use leads to adverse mental health	Threatening factors for mental health and well-being		

## RESULTS

### Characteristics of included articles

The included articles (*N* = 55) had a year range from 2015 to 2020. All articles were written in English and conducted in 28 countries from five continents: Asia (*n* = 33); North America (*n* = 12); South America (*n* = 1); Europe (*n* = 8); and Africa (*n* = 2). The articles included a variety of HE study populations and settings. The most common concept of the Internet use described was addictive or problematic Internet use. In the areas of health addressed, mental health issues were the most investigated (Supplementary File S2).

### Thematic findings

**Fig. 2:**
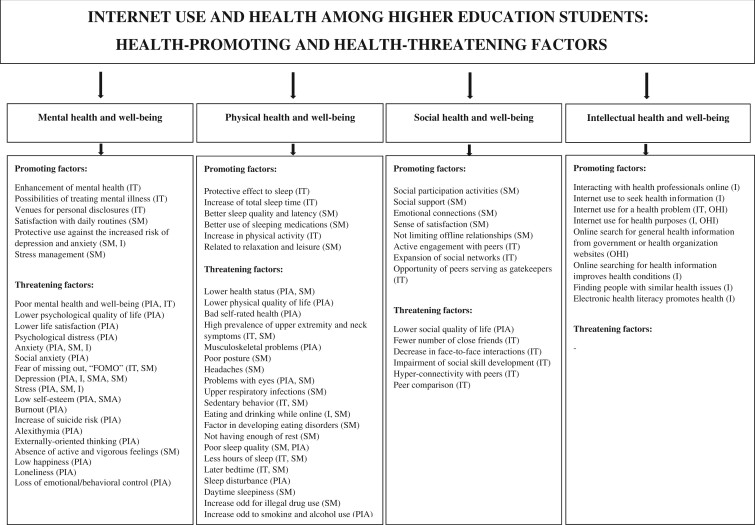
Health-promoting and Health-threatening Internet use. I, Internet use; IT, Internet-enhanced technology use; OHI, online health information-seeking behaviour; PIA, problematic or addictive Internet use; SMA, social media addiction; SM, social media use

The review identified two themes amongst the included articles. ‘Health-promoting Internet use’, included factors promoting mental, physical, social and intellectual health and well-being, and ‘Health-threatening Internet use’, contained factors threatening mental, physical and social health and well-being. The evidence was larger in the latter theme (Figure 2).

### Health-promoting Internet use

Factors promoting *mental health and well-being* included Internet-enhanced technology use and social media use. This category combined evidence on the enhancement of mental health, possibilities of treating mental illness (Lattie *et al.*, 2019) and satisfaction with daily routines (Austin-McCain, 2017). Also, information on better stress management related to relationships and work (Saini *et al.*, 2020).

*Physical health and well-being* category approached Internet use as social media use or Internet-enhanced technology use. Articles included information on the protective effect to sleep (Orzech *et al.*, 2016) and better sleep quality (Xu et al., 2016). Additionally, with an increase in physical activity levels (Wong, 2017) and with relaxation and leisure (Austin-McCain, 2017).

Health-promoting Internet use within *social health and well-being* approached Internet use mainly as social media use. Evidence about social participation activities (Austin-McCain, 2017) and social support were addressed (Mahapatra and Schatz, 2015). Also, active engagement with peers and expansion of social networks (Lattie *et al.*, 2019) were expressed.

*Intellectual health and well-being* contained Internet use or online health information-seeking behavior. This category included information on interacting with health professionals online (Asibey *et al.*, 2017; Lattie *et al.*, 2019), using the Internet for health purposes or to seek health information (Asibey *et al.*, 2017; Bati *et al.*, 2018; Levin *et al.*, 2020; Tariq *et al.*, 2020; Schwartz and Richardson, 2015). Evidence on how electronic health (e-health) literacy is promoting general student health was included (Britt *et al.*, 2017).

### Health-threatening internet use

Factors threatening *mental health and well-being* approached Internet use mainly from the problematic/addictive perspective including evidence with broad concept of poor mental health and well-being (Tangmunkongvorakul *et al.*, 2019; Tenzin *et al.*, 2018; Zhou et al., 2020; Hou *et al.*, 2019; Lattie *et al.*, 2019). Some of the articles also specified the factors in more detail, for example: distress (Al-Gamal *et al.*, 2015; Mamun *et al.*, 2020; Gedam *et al.*, 2017); depression (Khalil *et al.*, 2016, Othman and Lee, 2017, Peterka-Bonetta *et al.*, 2019; Younes et al., 2016; Iwamoto and Chun, 2020; Gedam *et al.*, 2017; Tao *et al.*, 2017; Asibong *et al.*, 2020; Chupradit *et al.*, 2020; Haand and Shuwang, 2020; Pang, 2020; Visnjic *et al.*, 2018); anxiety (Younes *et al.*, 2016; Campisi *et al.*, 2017; Iwamoto and Chun, 2020; Asibong *et al.*, 2020; Panova *et al.*, 2020; Gedam *et al.*, 2017); stress (Younes *et al.*, 2016; Campisi *et al.*, 2017; Liu *et al.*, 2017; Iwamoto and Chun, 2020; Unsar *et al.*, 2020); social anxiety (Weinstein et al., 2015); fear of missing out or FOMO (Lattie *et al.*, 2019; Pang, 2020); low happiness (Kitazawa *et al.*, 2019) and increase in suicide risk (Alpaslan *et al.*, 2015; Kurt, 2015; Poorolajal *et al.*, 2019).

Almost all articles addressing factors threatening *physical health and well-being* viewed Internet use as problematic/addictive or as social media use. This category included findings about lower health status (Jairoun and Shahwan, 2020; Kawyannejad *et al.*, 2019; Mohammadbeigi *et al.*, 2016a); a high prevalence of upper extremity and neck symptoms (Kalirathinam *et al.*, 2017; Rahman *et al.*, 2020). Behavioral aspects concerning Internet use while sedentary (Kalirathinam *et al.*, 2017) were also identified. Having fewer hours of sleep at night (Orzech *et al.*, 2016; Nasirudeem *et al.*, 2017; Mohammadbeigi *et al.*, 2016b; Thakur *et al.*, 2017; Whipps et al., 2018; Wang et al., 2020) was distinguishable. Increased odds of illegal drug use (Fogel and Shlivko, 2016) and smoking and alcohol use were also found (Tao *et al.*, 2017).

Health-threatening Internet use within the context of *social health and well-being* approached Internet use mainly from the Internet-enhanced technology perspective. This category included information on lower health-related quality of life in the social domain (Chern and Huang, 2018), fewer numbers of close friends (Lee *et al.*, 2016), hyper-connectivity with peers and peer comparison (Lattie *et al.*, 2019).

### A summary of the thematic findings

In summary, the findings indicated that Internet use among the HE student population is both health-promoting and health-threatening. Health-promoting Internet use provided beneficial health factors for the main aspects of personal health and wellbeing. On the contrary, health-threatening Internet use demonstrated that certain factors were risks, and threatened the health and wellbeing elements. The concepts used within these two findings are summarized in Supplementary File S3.

## DISCUSSION

Our study found that Internet use is associated with health from health-promoting and health-threatening dimensions. Factors promoting or threatening mental, physical, social and intellectual health and well-being were expressed. Furthermore, some of the health and well-being factors were bidirectional, belonging to both dimensions with different manners of approaches. For example, within the category of physical health and well-being, Internet use was associated with a protective effect for sleep (health-promoting) and with poor sleep quality (health-threatening). In general, the evidence of health-threatening Internet use was more prominent than evidence of health-promoting Internet use. A reason for this could be that the research on potential problems of excessive Internet use and addiction has increased considerably in recent years; the presence of Internet addiction and its associated behaviors, have been highlighted since the early 1990s (Shek *et al.*, 2013).

Evidence on HE students’ health-promoting Internet use accumulated mostly to categories of social and intellectual health and well-being. The promoting factors in social health and well-being identified issues, such as social participation activities and social support through social media. Hence, according to Bekalu *et al.* (2019), a routine social media use, meaning using social media within daily routines and responding to shared content, is in positive terms associated with social well-being. As students spend time social networking, they also develop relationships that can result in meaningful socio-psychological resources, supporting positive health behaviors (Paige *et al.*, 2017). Currently, social media is considered popular among HE students, especially networking sites such as Instagram, Facebook and Twitter, as well as multimedia messaging apps like Snapchat and the online video-sharing platform YouTube, which are used alongside different gaming sites, blogs and podcasts (Bragdon and Dowler, 2016; Lien *et al.*, 2018; Sutherland *et al.*, 2018; The Knight Foundation, 2020). Results in intellectual health and well-being suggest that HE students use the Internet for health purposes and to interact with health professionals online. Thus, the Internet enables easy accessibility to online health service by providing communicating channels with care providers and possibilities to receive care at home (Young and Nesbitt, 2017). Evidence on e-health literacy’s health-promoting aspect was also included. It comprises using electronic sources to address or resolve health problems with the proficiency to search, obtain, understand and evaluate health information (Yang *et al.*, 2017). However, health information-seekers are worried about obtaining deceptive material and exploring risk-promoting messages online (Mou *et al.*, 2017) – thus indicating the need for the activity of providing accurate health information in the online platforms where HE students operate, for example, in social media.

Results on HE students’ health-threatening Internet use indicated that factors threatening mental and physical health and well-being were the most comprehensive. Some factors were expressed broadly, and some in more detail. Most of the evidence supporting the factors threatening mental and physical health and well-being were from Asian countries and focusing on problematic or addictive Internet use. As reviewed by Li *et al.* (Li *et al.*, 2018), the prevalence of Internet addiction disorders (IAD) is greater in Asia than in Europe. For instance, in China, Internet addiction is acknowledged as an official disorder (Kuss and Lopez-Fernandez, 2016). HE students, together with high school students, are known to be more vulnerable to these addictions compared with other student groups (Turnbull *et al.*, 2018), although children and adolescents are also becoming increasingly addicted to playing Internet games (Bener *et al.*, 2016). Overall, currently, the addictive or problematic form of Internet use is viewed as a notable growing health problem among HE students, affecting their mental and physical health (Kuss and Lopez-Fernandez, 2016; Shao *et al.*, 2018; Fernandes *et al.*, 2019). Conclusively, health-threatening Internet use demonstrates the necessity of preventive actions, such as focused health-promoting social marketing actions, to avoid risky behaviors from occurring among students.

This comprehensive scoping review captured the majority of the relevant literature on the association of Internet use and health. A systematic, rigorous and transparent methodology was used based on a methodological framework. The results provided a broad overview of the topic in accordance with the research question. The results have less depth because the literature is vast and complex (Arksey and O'Malley, 2005; Peterson *et al.*, 2017). The majority of the articles in this review were from Asian countries. This might be because in the Asia-Pacific regions, the Internet use related addiction is viewed as a current concern in public health amongst young adults (Tang *et al.*, 2017). Limitations of this research include the use of articles written only in English, Finnish or Swedish. Also, as typical for scoping reviews, the quality of included articles was not examined (Arksey and O'Malley, 2005). The judgment of the trustworthiness within the value and relevance of the articles included needs to be taken into account, in accordance with the aim of this review. Further, the results of this scoping review can be utilized in planning a future systematic review that exploits a quality appraisal (Munn *et al*., 2018).

## CONCLUSION

This scoping review characterizes and describes the evidence on the association between Internet use and health among HE students. Internet use is health-promoting mostly for social and intellectual health and well-being, and health-threatening primarily for mental and physical health and well-being. This bifurcation should be taken into account in promoting HE students’ health. We hope that the findings of our review can assist ongoing research to further clarify and enhance the association between Internet use and health.

## SUPPLEMENTARY MATERIAL

Supplementary material is available at *Health Promotion International* online.

## AUTHORS’ CONTRIBUTIONS

H.R. was responsible for the literature searches and the data analysis via the thematic analysis method. K.J. and H.R. conducted the dual-review process. K.J., M.S. and H.T. made critical revisions to the paper. M.S. and H.T. verified all the processes in conducting this scoping review and supervised the study.

## FUNDING

This research was funded by the University of Eastern Finland’s Doctoral School, the Doctoral Programme in Health Sciences and the Department of Nursing Science. 

Conflict of Interest: The authors declare that they have no conflict of interest.

## Supplementary Material

daab007_Supplementary_Data
