# Supplementary material for: Internet use and health in higher education students: a scoping review
Source: Health Promot Int. 2021 Mar 19;36(6):1610–20. doi: 10.1093/heapro/daab007 (PMC8699394; doi:10.1093/heapro/daab007)
Supplement: daab007_Supplementary_Data [file daab007_supplementary_data.zip › Supplementary file 1.docx]

Supplementary file 1. The inclusion and exclusion criteria for the title, abstract and full-text review phases of study selection using the PCC framework (Joanna Briggs Institute 2019)

| Category | Inclusion criteria | Exclusion criteria |
| --- | --- | --- |
| Population | **Higher education student**  Higher education student also referred as:  - University student  - University of applied sciences student  - College student  - Polytechnic student  - Tertiary student |  |
| Concept | **Internet use**  Internet use (problematic, excessive or addictive use)  Internet use by time  Online activities (chat/messaging, studying, working, e-mailing, information searching, watching TV or videos, or movies or sports or adult entertainment, listening to music, reading books and magazines, gaming)  Social media use, social networking sites use  Enabled by technology (information and communication technology (ICT), smart/ mobile device) | Internet use guided by professionals (health treatment or health intervention) |
|  | **Health**  Holistic concept of health (holistic health includes physical, mental, social, spiritual and emotional dimensions (Eberst 1984) |  |
| Context | **Association on Internet use and health among higher education students**  Any geographic location  All types of study designs  Articles published from 2015 to 2019 | Protocols, reviews |
