# Supplementary material for: Internet use and health in higher education students: a scoping review
Source: Health Promot Int. 2021 Mar 19;36(6):1610–20. doi: 10.1093/heapro/daab007 (PMC8699394; doi:10.1093/heapro/daab007)
Supplement: daab007_Supplementary_Data [file daab007_supplementary_data.zip › Supplementary file 2.docx]

Supplementary file 2. Data extraction of reviewed articles

| **Author information** | **Study /article objective** | **Study /article design and sample** | **Outcome measures**  ***(Internet use measurement described)*** | **Main findings** |
| --- | --- | --- | --- | --- |
| Al-Gamal et al., 2016  (Jordan, Asia) | To measure the prevalence of Internet addiction (IA) and its association with psychological distress and coping strategies | Survey  N=587 | Perceived Stress Scale (PSS), Coping Behavior Inventory Scale  *(Internet Addiction Test (IAT)* | IA was associated with high psychological distress. Students who used problem solving as a coping strategy, were more likely to experience a lower level of IA |
| Alpaslan et al., 2015  (Turkey, Europe) | To investigate the relationship between problematic Internet use (PIU) and loneliness, alexithymia and probability of suicide | Survey  N=328 | UCLA loneliness scale (UCLA), the 20-item Toronto Alexithymia Scale (TAS-20), Suicide Probability Scale (SPS)  *(Young Internet Addiction Test (YIAT)* | PIU was found to be associated with loneliness and probability of suicide in areas of hopelessness, negative self-evaluation and hostility. In addition, PIU was associated with alexithymia in difficulties in identifying and describing feelings as well as with externally oriented thinking |
| Asibey et al, 2017  (Ghana, Africa) | To investigate the use of the Internet for health purpose | Survey  N=650 | Health purposes and use of online health information  *(Internet use was measured with questions regarding amount of Internet use, Internet access and type of Internet use)* | Internet is used for health purposes or to seek health information. Health purposes as for interacting with health professionals. Online health information for decision making and for actions concerning health, such to lifestyle change  The search for health information improved students’ health conditions |
| Asibong et al., 2020  (Nigeria, Africa) | To assess the psychological effects of Internet/social media usage | Survey  N=418 | General Health Questionnaire‑28  *(Internet Addiction Test)* | Respondents with moderateto severe forms of Internet addiction had significantly lower mean depression and anxiety scores compared with those with mild or no form of addiction |
| Austin-McCain 2017  (USA, North America) | To examine the association between social media use and healthy living habits. In addition to examine social media use association with greater satisfaction with the daily routines | Survey  N=164 | Healthy living habits  were measured by the following activities related to healthy living: fitness and exercise, healthy eating, relaxation, personal self- development, participation in leisure activities, and social participation with family and friends  Satisfaction with daily routine was measured by asking the participants about their overall satisfaction with their daily routines  *(Social media use was measured by asking to select the option that best described social media use)* | Social media use at least 6 days a week is positively related to relaxation, leisure and social participation activities. Individuals with greater social media use reported being satisfied with their daily routines  No significant association was found between social media use and other healthy habits, such as fitness and healthy eating |
| Bati et al., 2018  (Turkey, Europe) | To determine the factors (incl. Internet use, health information seeking online) affecting cyberchondria and health anxiety levels | Survey  N=867 | Health anxiety symptoms were measured using the Health Anxiety Inventory questions  Cyberchondria was measured with Cyberchondria Severity Scale (CSS)  *(Internet use was measured with asking what technology is used to access the Internet. In addition, online health information seeking behavior was examined)* | When students had a health problem, they tried to understand the problem by seeking health information on the Internet  In the presence of a health problem of health anxiety and cyberchondria, health related online searching increased  Half of the students were skeptical of trusting the online health information |
| Britt et al., 2017  (USA, North America) | To examine what EHealth literacy (intentions toward using the Internet for health information) reveals about health behaviors | Survey  N=422 | eHealth Literacy Scale  Behavior was measured with a single item for each of the 8 health areas identified from the American College Health Association (ACHA)  *(Intentions toward using the Internet for each of the 8 areas identified from the ACHA were measured using a theory: “Theory of Planned Behavior”)* | Electronic health literacy is significantly correlated with an individual’s general health, exercise regime, sleep, getting vaccines, and maintenance of sexual health, a balanced diet, stable friendships, and a lifestyle free of harmful substances  Current use of online health resources had a moderate relationship for seeking out information in the following areas: a balanced diet, safe sex practices, exercise, and vaccines |
| Campisi et al., 2017  (USA, North America) | To examine whether virtual social network size (=number of Facebook friends) was significantly related to the rate of upper respiratory infection (URI) and if anxiety/stress modifies this interaction. In addition to examine, if other components of Facebook use (=minutes/day, log-ins/day, behavior) were associated with stress and/or increased risk of URI | Survey  N=89 | 4-item Global Measure of Perceived Stress, Satisfaction with Life Scale, Multidimensional Scale of Perceived Social Support  *(Facebook use was measured with examining technology use in general)* | Virtual social network size was significantly related to the rate of URI  Individuals who demonstrate anxiety/stress regarding their Facebook use are more likely to log-in to Facebook |
| Chupradit et al., 2020  (Thailand, Asia) | To study the relationship between Internet usage behavior and depression in the first-year undergraduate students who stay in the dormitories in Chiang Mai University | Survey  N=500 | The Patient Health Questionnaire-9 (PHQ-9)  *(Internet Addiction Test (IAT)* | Internet usage and depression are moderately positively correlated |
| Fogel and Shlivko, 2016  (USA, North America) | To study the association of watching reality TV programs or following a reality TV character on Twitter with illegal drug use or prescription drug misuse | Survey  N=576 | Two questions related to illegal drug use or prescription drug misuse were used with yes/no choices  *(TV related questions were based on social cognitive theory, parasocial interaction theory and cultivation theory. In addition, one Twitter related question was used with yes/no choice)* | Following a reality TV character on Twitter had statistically significant increased odds for illegal drug use |
| Chern and Huang, 2018  (Taiwan, Asia) | To examine the associations between Internet addiction (IA) and health-related quality of life (HRQOL) | Survey  N=1452 | World Health Organization Quality of Life assessment short version (WHOQOL-BREF)  *(Chen Internet Addiction Scale (CIA)* | Students with IA had significantly lower HRQOL in physical, psychological, social, and environment domains.  Students showing interpersonal and health problems owing to Internet use also reported lower social HRQOL |
| Gedam et al., 2017  (India, Asia) | To estimate the prevalence of internet addiction, understand the pattern of internet use, and to determine the association between psychopathology and internet addiction | Survey  N=846 | The Mental Health Inventory  *(The Internet Addiction Test (IAT).)* | The psychiatric symptoms such as anxiety, depression, loss of emotional/behavioral control, emotional ties, life satisfaction, psychological distress, and lower psychological well‑being had significant association with IA |
| Haand and Shuwang, 2020  (Afghanistan, Asia) | To examine the relationship between social media addiction and depression | Survey  N=384 | Depression was measured by theCentre for Epidemiologic Studies Depression Scale (CES-D)  *(Kimberly Young’s Internet Addiction Test (IAT) was applied and replicated for measuring social media addiction)* | Social media addiction has a positive correlation with depression and depression significantly predicts social media addiction, the higher the student addiction level, the greater his/her depression level is |
| Hou et al., 2019  (China, Asia) | To examine the relations of social media addiction to mental health | Survey and Intervention  N=273 | General Health Questionnaire (GHQ-30), Self-esteem Scale (Chinese version)  *(the Bergen Social Media Addiction Scale (BSMA)* | Social media addiction is negatively associated with students’ mental health, partly through lowering individuals’ self-esteem |
| Iwamoto and Chun, 2020  (Hawaii) | To determine the relationship between social media use and its impact on depression, anxiety, and stress | Survey  N=181 | The DASS-21  *(The DASS-21 includes questions on social media use)* | A positive correlation between the hours of social media use with depression, anxiety, and stress was found |
| Jairoun and Shahwan, 2020  (United Arab Emirates, Asia) | To explore health issues in relation to social media use | Survey  N=444 | Health issues were questioned with eleven questions to indicate how often they had experienced a number of specific health complaints in the past week  *(Social media usage was questioned with how many hours of social media is used during the past week)* | The majority of respondents were active on social media and that half could be categorised as having a poor health status |
| Kalirathinam et al., 2017  (Malaysia, Asia) | To determine the association between the usage of the smartphone as the risk factor for the prevalence of upper extremity and neck symptoms | Survey  N=250 | Physical potions were questioned by upper extremity and neck symptom related questions. In addition, question related to smartphone use positions were included  *(Internet use (incl. e-learning, music, video, gaming) was assessed with smartphone use related questions)* | A high prevalence of upper extremity (shoulder, elbow and lower arm, and hand) and neck symptoms in various positions were noted when using the smartphone  Majority of participants frequently used smartphone while sitting |
| Kawyannejad et al., 2019  (Iran, Asia) | To evaluate general health status and to determine the predictive role of variables such as cell phone usage, sleep quality, internet addiction (IA) and social networks addiction | Survey  N=321 | General Health Questionnaire, Pittsburgh Sleep Quality Index  *(Internet Addiction Test, Social Network Addiction Questionnaire, Cell Phone Overuse Scale)* | Students who had overuse of cell  phone had lower general health status than the reference category (students with cell phone little use)  No significant relationship between age, levels of Internet and social networks addiction and the general health score were demonstrated |
| Khalil et al., 2016  (Saudi-Arabia, Asia) | To investigate the prevalence of Internet Addiction (IA) and its association with mental health | Survey  N=147 | The Center for Epidemiologic Studies Depression Scale (CES-D)  (Internet Addiction Test (IAT), Arabic version) | A significant correlation was found between IA, time spent on the Internet, and depressive symptoms |
| Kitazawa et al., 2019  (Japan, Asia) | To clarify the relationship between happiness and Internet use | Survey  N=1258 | HIS Scale by Hitokoto  *Internet addiction test (JIAT), Japanese version. In addition, Internet usage time, most-accessed internet applications and use of social networking sites were questioned)* | The high happiness group had a significantly lower PIU tendency, a better sleep state and a greater number of Twitter follows compared to the low happiness group  Low happiness group used the Internet for longer periods of time compared to high happiness group |
| Kurt, 2015  (Turkey, Europe) | To identify the factors in suicide risk among college students by examining the direct and indirect effects of drug use, internet addiction, gender, and alcohol use on suicide risk. | Survey  N=975 | the University Form of Risk Behaviors Scale (UFRBS)  *(Internet Addiction Scale (IAS)* | Internet addiction was found to lead to an increase in suicide risk  Increase in alcohol use leads to an increase in Internet use |
| Lattie et al., 2019  (USA, North America) | Technology and college student mental health: challenges and opportunities | Perspective article | Mental health perspective based on research results  *(Use of personal computing technologies (including social media use) and technology-enabled mental health services)* | The rise in mental health symptoms with the rise of personal computing technologies (including social media use), have suggested that time spent on these types of technologies is correlated with poor mental health. There is although a controversy, some evidence suggests that social media use, as Facebook use, is only harmful to mental health when it is passive viewing of other people’s posts as opposed to more active engagement in social connections, for example social support networks  The same technologies also offer several opportunities for the enhancement of mental health and the treatment of mental illness. Technology-enabled mental health services offer the possibility to expand treatment options and reduce barriers to mental health services |
| Lee et al., 2019  (Malaysia, Asia) | To evaluate the relationship between Facebook usage and/or mobile texting through a focus on excessive Facebook use and excessive mobile texting as they relate to health impacts | Survey  N=188 | WHO-Five Well-being Index (WHO-5), Cut down, Annoyed, Guilty, Eye-opener (CAGE)  *(The questionnaire included questions on texting habits and items assessing Facebook usage habits. Also, questions related to dependence on mobile texting and Facebook use were included)* | Fewer number of close friends, checking updates on the Facebook walls of friends, and the absence of active and vigorous feelings during Facebook use were significant predictors of excessive Facebook use |
| Lebni et al., 2020  (Iran, Asia) | To vestigate internet addiction and its effects on the mental health | Survey  N=447 | Goldberg General Health Questionnaire 28  *(Young’s Internet Addiction Test)* | Excessive internet usage leads to anxiety, depression, and adverse mental health |
| Levin et al., 2020  (Usa, North America) | To evaluate the feasibility and acceptability of a widely available mindfulness health app for college students waiting to receive services at a college counseling center | Survey and InterventionN =23 | Counseling center assessment of psychological symptoms-34 item version (CCAPS-34)  Mental health continuum–short form (MHC-SF)  Five facet mindfulness questionnaire (FFMQ)  Valuing questionnaire (VQ)  System usability scale (SUS)  *(Participants self-reported how many days they used the smart phone app in the past two weeks)* | Students provided high satisfaction ratings with the app and reported regular use of the app. Results provided very preliminary support for the potential efficacy of the app, particularly for depression, anxiety, and general distress |
| Liu et al., 2017  (USA, North America) | To investigate whether students’ perceived levels of stress were associated with the sentiment and emotions of their tweets | Survey and a Twitter streaming application to retrieve tweets  N=121 | Psychometric measures of stress were questioned by overall levels of stress and sources of stress for the last 7 days  *(Participants tweets from the Twitter were retrieved.)* | Tweet sentiment was associated with participants’ future survey about their emotions and stress  Higher levels of stress and emotion of fear were associated with a greater percentage of negative sentiments and percentage of tweets related to fear. In addition, perceived level of stress was also positively associated with the percentage of tweets with love and hope |
| Mahapatra and Schatz, 2015  (USA, North America) | To obtain information about the use of social networking sites (SNSs) and examine if there is any relationship between the use of SNSs, students perceived social support in their lives, and their perceived general well-being | Survey  N=190 | The General Well-Being Schedule scale. Social support was assessed by questions related to perceived social support (both offline and online) and relationships maintained online  *(Internet use was assessed with questions on usage of SNS(s), a three-item scale developed by Valkenburg et al. 2006. In addition, questions on the level of self-disclosure or how much they shared on social network sites was questioned)* | Students felt emotionally connected and felt an overall sense of satisfaction when they connected friends (=important resource of social support) on SNSs  The reliance for online interactions did not discourage or limit students offline relationship experience |
| Mamun et al., (2020)  (Bangladesh, Asia) | To examine the prevalence of problematic Internet use (PIU) and its associated risk factors including sociodemographic variables, Internet use behaviors, and other psychological variables including loneliness, self-esteem, and psychological distress | Survey  N=605 | Rosenberg's Self-Esteem Scale, the UCLA Loneliness Scale, and the General Health Questionnaire  *(The Internet Addiction Test (IAT)* | Loneliness and psychological distress were positively correlated with PIU.  Individuals who are lonely, have low self-esteem, and experience psychological distress were more likely to experience PIU |
| Mohammadbeigi et al., 2016a  (Iran, Asia) | To find the relationship be-tween self-rated health (SRH), internet addiction, and determinants of SRH and internet addiction (IA) | Survey  N=254 | SRH was measured by three different questions on areas of rating general health status and estimating own health  *(Young’s Internet Addiction Questionnaire (YIAQ)* | E-mailing and scientific websites use were related with good self-rated health while IA is associated with bad self-rated health |
| Mohammadbeigi et al., 2016b  (Iran, Asia) | To identify the relationship between sleep qualities due to overuse of mobile cell-phones and engagement in social networks. | Survey  N= 355 | Pittsburgh Sleep Quality Questionnaire (PSQI)  *(Cell-Phone Over-Use Scale (COS) and the use of mobile cell-phone’s social networking apps)* | Most of the students reported using social networking applications in the last hours of night or after midnight instead of having rest  Male students were more addicted to smart cellphones and suffered from poor sleep quality. |
| Nasirudeem et al., 2017  (Singapore, Asia) | To examine the relationships between self-reports of social media usage and daytime sleepiness | Survey  N=969 | Cleveland Adolescent Sleepiness Questionnaire(CASQ), modified version  *(Social media usage was assessed with questions on the amount of time spent on each social media’s social networking site (SNS) and reasons for the use )* | Students who spent more time on SNS by staying up late at night experienced greater daytime sleepiness  The longer the students stayed up late at night to spend time on SNS, the lesser were their hours of sleep |
| Orzech et al., 2016  (USA, North America) | To investigate whether self-reported sleep patterns are associated with digital media use | Survey and Diary  N=254 | Total Time in Bed (TIB), Total Sleep Time (TST, Bedtime (B), Sleep Onset Latency (SOL), Wake After Sleep Onset (WASO)  *(Questions related to digital media activities)* | Digital media quantity predicted later bedtime and reduced total sleep time  More diversity in digital media activities was associated with increased total sleep time. Especially using multiple media in the 2 h before bedtime had a protective effect on sleep |
| Othman and Lee, 2017  (Malaysia, Asia) | To examine the internet addiction (IA) and its association with depression and anxiety | Survey  N=267 | The Hospital Anxiety and Depression scale (HADS)  *(The Internet Addiction Test (IAT). In addition questions on the duration of use in hours, device for use and form of use (e.g social networking, chatting, surfing, games, e-mailing, downloading, or shopping) on the Internet was questioned)* | Depression was significantly associated with IA, while anxiety was not |
| Pang, 2020  (China, Asia) | To offer a deeper comprehending of the interrelationships between distinct patterns of WeChat interactions, upward social comparison, depressed mood, and the fear of missing out among university students | Survey  N=318 | Upward social comparison rating scale  Fear of missing out scale by Przybylski et al. 2013  Depressive mood by Patient-Reported Outcomes Measurement System (PROMIS)  *(WeChat (=social media) use* | WeChat use is associated with greater depressive symptoms and fear of missing out |
| Panova et al., 2020  (Spain, Europe; United States, North America; Colombia, South America) | To explore 5 popular uses of the smartphone–messaging, browsing the Internet, posting social content, reading social content, and playing games–how they relate to anxiety and depression scores | Survey  N=1709 | Mood and Anxiety Symptoms Questionnaire (MASQ)  (Mobile Related Experiences Questionnaire (CERM)) | In the USA, game playing contributed to anxiety scores and reading social content was a protective use against anxiety scores  In Spain, browsing the Internet was the only specific use that contributed to anxiety scores. The use of posting social content showed a protective use against depression scores  In Colombia, no specific smartphone use was a contributing variable regarding anxiety scores and only game playing had a significant role in depression scores as a protective factor |
| Peterka-Bonetta et al., 2018  (China, Asia;  Germany, Europe) | To investigate the relationship between depression and Internet Use Disorder (IUD) and between burnout and IUD | Survey  N=266 | The Beck Depression Inventory II (BDI-II), the Maslach Burnout Inventory-General Survey (MBI-GS)  *(The Generalized Problematic Internet Use Scale (PIUS2). In addition, Internet use for private and business purpose were questioned)* | Associations between burnout, depression and IUD was observed |
| Poorolajal et al., 2019  (Iran, Asia) | To assess the prevalence of problematic Internet use (PIU) and associated predisposing factors and complications | Survey  N=4261 | General Health Questionnaire (GHQ)  *(PIU-15 questionnaire)* | Students suffering from PIU were more likely to experience health-threatening conditions such as poor general health and increased risk of suicidal behaviors |
| Rahman et al., 2020  (United Arab Emirates, Asia) | To nvestigate the effects of social media use on health | Survey and Guided interviews N=300 | The surveys included questions on general health (eight questions)  The interview included questions on General Health (two questions), and self-regulation (two questions)  *(Social media use was questioned in the survey and in the interviews)* | Social media use delayed bedtime, and affected to the quantity and quality of sleep  Social media use reduced physical activity and is a factor in developing eating disorders  As result of social media use, eye strain, fatigue, neck/shoulder pain, headaches, and poor posture were experienced |
| Qader et al., 2015  (Kuala Lumpur, Asia) | To explore the nutritional habits of students, especially those who use internet heavily | Survey N=361 | Nutritional habits were measured with questions related to eating breakfast and dinner daily and fruit and vegetable intake. In addition, questions about drinking water, carbonated drinks and energy drinks were measured  *(Internet use was measured with questions assessing hours of Internet use. In addition, questions about Facebook account existence and Facebook use per day)* | The association between taking dinner daily and using Internet was significant, as well as drinking cans of carbonated drinks and using the Internet  Over half of students reported eating something while using Internet. In addition, almost half of students reported playing with their mobile while eating  Students are using Internet heavily and this may affect their nutritional habits and daily lifestyle choices |
| Saini et al., 2020  (India, Asia) | To understand how the time spent on social networking is affecting the quality of life | Survey  N=220 | Quality of life was measured wtih questions of physical state(6 items), mental state (10 items), stress evaluation (10 items),life enjoyment (10 items), the perceived overall quality of life(11 items), and overall impression about all the five domains(5 items)  *(Young’s Internet usage questionnaire and questions regarding usage of social networking sites’ (SNSs), number and name of the most frequently visited sites, and the three best and worst things about the Internet)* | Daily users of the SNSs were better able to handle the stress related to relationships and work  Fear of missing out, maladaptive cognitions, and psychiatric distress significantly predicted SNS addiction |
| Schwartz and Richard, 2015  (Canada, North America) | To assess the use of internet-enabled technology for seeking health information and resources and to examine attitudes related to the use of the internet to deliver evidence-based interventions for the prevention and treatment of overweight and obesity. | Survey  N=500 | The Douglas College Student Health and Wellness Survey  *(Internet use was assessed questions concerning their health-seeking behaviors and preferences regarding the use of internet-enabled technology)* | Overweight/obese students were aware of their weight status and receptive to using the internet for health purposes  When seeking general health information, majority of students would use websites, as government or health organizations websites, as sources |
| Tangmunkongvorakul et al., 2017  (Thailand, Asia) | To examine the relationship between smartphone use and psychological well-being. | Survey  N=800 | the Flourishing Scale (FS)  *(The Young Diagnostic Questionnaire for Internet Addiction)* | Students with excessive levels of smartphone use had lower scores on the social-psychological well-being than those who did not use smartphone excessively |
| Tao et al., 2017  (China, Asia) | To assess the multiplicative and additive interactive effect of problematic mobile phone use (PMPU) and depressive symptoms with alcohol use. | Survey  N=2376 | A Self-rating Depression Scale (SDS), Young Risk Behavior Surveillance System (YRBSS)  *(The Self-rating Questionnaire for Adolescent Problematic Mobile Phone Use (SQAPMPU)* | Students who reported having an Internet addiction had a higher rate of depressive symptom  Students who were current smokers and Internet addicts had a higher percentage of PMPU. Also, students with PMPU were more likely to use alcohol |
| Tariq et al., 2020  (Pakistan, Asia) | To assess the patterns of Internet use and eHealth literacy levels and to examine the association of the eHealth literacy levels with physical activity levels and dietary supplement intake | Survey  N=505 | The eHealth literacy scale (eHEALS)  Health behaviors were questioned with questions on physical activity and the use of dietary supplements  *(The Internet use patterns were explored by asking questions on the frequency of Internet use for browsing health information and common health-related topics. Also the frequency of using Google and other social media platforms as a venue for seeking health information was questioned)* | The most common type of health-related information that was searched by the participants on the Internet was that related to maintaining a healthy lifestyle  Participants with high eHEALS scores were those who used the internet frequently for finding people with similar health issues  The use of specific social media platforms was not associated with the perceived eHealth literacy levels. Neither the frequency of physical activity nor the dietary supplement use was associated with the eHealth literacy of the participants |
| Tenzin et al., 2018  (Bhutan, Asia) | To understand the patterns, prevalence, and the associated factors for internet addiction (IA) | Survey N=719 | The General Health Questionnaire  *(Internet Addiction Test (IAT)* | An association between IA and mental wellbeing was observed |
| Thakur et al., 2017  (India, Asia) | To determine the level of internet addiction (IA) and various behavioral aspects (incl. health-related problems) related to it | Survey  N=425 | Questions related to health were questioned (not described in more detail)  *(The Young’s Internet Addiction Test.)* | Students who had IA had more sleep disturbance and problems with eyes  Students having IA had more musculoskeletal problems i.e. pain around the region of neck, shoulders, waist, finger and wrist |
| Unsar et al., 2020  (Turkey, Europe) | To determine the relationship between Internet use and stress levels in university students and the factors affecting them | Survey  N=433 | The DASS Stress Sub-scale (SS)  *(The Online Cognitive Scale (OCS)* | Stress levels and problematic Internet usage levels among students increased as the duration of their Internet use increased |
| Visnjic et al., 2018  (Serbia & Italy, Europe) | To explore the manner and intensity of the use of mobile phones and examine its effects on certain mental health aspects | Survey  N=785 | Depression Anxiety Stress Scale (DASS 42)  *(Internet use was assessed by questions on time spent on the internet, time spent using various applications, and time spent playing game.)* | Students with anxiety symptoms spend less time browsing the Internet  Depressed students make fewer calls and browses the internet less frequently on the mobile phone, but send more SMSs |
| Wong, 2017  (China, Asia) | To compare the players and ex-players’ frequencies and durations of staying outdoors, and walking/jogging before and during the time they played Pokémon Go, and to assess and compare the physical activity levels, including vigorous activities, moderate activities and walking, of players, ex-players and non-players of Pokémon Go | Survey  N=784 | The International Physical Activity Questionnaire (IPAQ)  *(Pokémon Go Internet gaming was measured by questions on time spent in playing Pokémon Go and time spent in staying outdoors, walking and/or jogging intentionally when playing the game, and before started playing the game)* | The players who were used to being sedentary benefited the most from Pokémon Go. Meaning, the players who never or rarely stayed outdoors or walked/jogged before would significantly stay outdoors more frequently as well as walking/jogging more often in order to play Pokémon Go  There was no significant difference in the overall physical activity levels and total physical activity between players, ex-players and non-players |
| Xu et al., 2016  (China, Asia) | To identify the relationship between WeChat use and sleep quality | Survey  N=1979 | Pittsburgh Sleep Quality Index (PSQI)  *(A screening instrument for using WeChat was used with yes/no choice. Also questions on how social media use effects on sleep quality was questioned)* | WeChat users have better sleep quality in subjective quality of sleep, sleep latency, use of sleeping medication, daytime dysfunction, and global PSQI among undergraduates than non-WeChat users |
| Wang et al., 2020  (China, Asia) | To examine the association between subjective sleep quality and problematic Internet use (PIU) | Survey  N=1040 | Pittsburg Sleep Quality Index (PSQI), Subjective Sleep Quality (SQQ)  (Young’s 20-item Internet Addiction Test (IAT)) | Poor subjective sleep quality was strongly associated with elevated odds of PIU over and above the contributions of poor psychological functioning, substance use, and physical activity |
| Weinstein et al., 2015  (Israel, Europe) | To assess Internet addiction (IA) and social anxiety | Survey N=240 | The Liebowitz Social Anxiety Scale  *(the Internet Addiction Test (IAT)* | A moderate positive association between IA and social anxiety was observed  Students with high levels of social anxiety do not use the Internet mainly for social purposes (e.g. social networking) necessarily |
| Whipps et al., 2018  (USA, North America) | To determine the association between presence and use of media devices at night-time | Survey  N=128 | Pittsburgh Sleep Quality Index (PSQI)  Height and weight measurements were done  *(Nighttime media*  *usage (NMU)* | A relationship exists between sleep behaviors and nighttime media usage. When nighttime usage (texting, social media, gaming, etc.) increases, overall incidence of sleep interruptions increases, and sleep quality tends to decrease |
| Younes et al., 2016  (Lebanon, Asia) | To assess relationships between potential Internet addiction (IA), insomnia, depression, anxiety, stress and self-esteem | Survey  N=600 | The Insomnia Severity Index, the Depression Anxiety Stress Scales(DASS21), the Rosenberg Self Esteem Scale (RSES)  *(The Young’s Internet Addiction Test.)* | A strong correlation between potential IA and anxiety, stress, and depression was observed  A decrease in self-esteem seems to be associated with potential IA |
| Zhou et el., 2020  (China, Asia) | To delineate the temporal dynamics of the association between problematic Internet use (PIU) and mental health issues among Chinese college students using an analytic approach that can effectively disaggregate the within-person and between-person effects | Survey  N=2329 | The Symptom Check-List-90 (SCL-90)  *(Problematic Internet (PIU) use was assessed using the 26-item Revised Chinese Internet Addiction Scale (CIAS-R)* | The contribution of PIU to college students’ mental health issues over time may be attributable to both the more stable, trait-like differences between individuals and the more statelike fluctuations within individuals. An association between PIU and mental health issues was found. |
