# Supplementary material for: Internet use and health in higher education students: a scoping review
Source: Health Promot Int. 2021 Mar 19;36(6):1610–20. doi: 10.1093/heapro/daab007 (PMC8699394; doi:10.1093/heapro/daab007)
Supplement: daab007_Supplementary_Data [file daab007_supplementary_data.zip › Supplementary file 3.docx]

Supplementary file 3. Summary of the concepts used within the thematic findings of “Health-promoting Internet use” and “Health-threatening Internet use” and definitions in the context of Internet use.

| **Concept** | **Definition in the context of Internet use** |
| --- | --- |
| ***Mental health and well-being*** |  |
| ***Health-promoting Internet use*** |  |
| Daily routines | Students with greater social media use report being satisfied with their daily routines (Austin-McCain, 2017). |
| Increased risk of depression and anxiety | High degree of Internet addiction may be protecting against the increased risk of depression and anxiety. T (Asibong et al., 2020) |
| Mental health | Technologies that use the Internet offer several opportunities for the enhancement of mental health (Lattie et al., 2019). |
| Mental illness | Internet and technology-enabled mental health services offer the possibility to expand treatment options for mental illnesses (Lattie et al., 2019). |
| Personal disclosures | Social media platforms, such as Facebook and Instagram, are increasingly seen as venues for personal disclosures (Lattie et al., 2019). |
| Stress management | Daily users of the SNSs were better able to handle the stress related to relationships and work (Saini et al., 2020) |
| ***Health-threatening Internet use*** |  |
| Active and vigorous feelings | Absence of active and vigorous feelings during Facebook use are significant predictors of excessive Facebook use (Lee et al., 2019). |
| Alexithymia | Problematic Internet use is associated with alexithymia, meaning inability to identify and describe feelings (Alpaslan et al., 2015). |
| Anxiety | Anxiety is associated with Internet addiction (Gedam et al., 2017; Younes et al., 2016). Students who demonstrate anxiety regarding their Facebook use are more likely to log-in to Facebook (Campisi et al., 2017). |
| Burnout | Internet use disorder is associated with burnout (Peterka-Bonetta et al., 2018). |
| Depression | Depression is associated with Internet addiction, Internet use disorder and higher time spent on the Internet (Gedam et al., 2017; ; Khalil et al., 2016, Othman and Lee, 2017, Peterka-Bonetta et al., 2018; Tao et al., 2017; Visnjic etl al., 2018; Younes et al., 2016). |
| Emotional/behavioral control | Loss of emotional/ behavioral control is associated with Internet addiction (Gedam et al,. 2017). |
| Externally-oriented thinking | Problematic Internet use is associated with externally-oriented thinking, meaning a tendency to focus on the concrete details of external events rather than of feelings (Alpaslan et al., 2015). |
| Fear of missing out, “FOMO” | Proposed negative effect of personal computing technology use (especially social media use) on mental health on the subject of fearing of social and information exclusion (Lattie et al., 2019). |
| Happiness | Students with lower happiness have a higher problematic Internet use tendency (Kitazawa et al., 2019). |
| Life satisfaction | Lower life satisfaction is associated with Internet addiction (Gedam, 2017). |
| Loneliness | Loneliness was positively correlated with problematic Internet use (Mamun et al., 2020) |
| Mental health and well-being | The rise of personal computing technologies (including social media use), have suggested that time spent on these types of technologies is correlated with poor mental health (Lattie et al., 2019). Students with excessive levels of smartphone use and Internet addiction have lower psychological well-being (Tangmunkong et al., 2017; Tenzin et al., 2018). |
| Psychological quality of life | Students with Internet addiction have significantly lower health-related quality of life in psychological domain (Chern and Huang, 2018). Lower psychological wellbeing is associated with Internet addiction (Gedam, 2017). |
| Psychological distress | Internet addiction is associated with high psychological distress. (Al-Gamal et al., 2016; Gedam et al., 2017). |
| Self-esteem | Social media addiction lowers students’ self-esteem (Hou et al., 2019). A decrease in self-esteem is associated Internet addiction (Younes et al., 2016). |
| Social anxiety | Internet addiction and social anxiety are associated. Students with high levels of social anxiety do not use the Internet mainly for social purposes (e.g. social networking) necessarily (Weinstein et al., 2015.) |
| Stress | Stress is associated with Internet addiction (Younes et al., 2016). Individuals who demonstrate stress regarding their Facebook use are more likely to log-in to Facebook (Campisi et al., 2017). Internet tweet sentiment (from the Twitter) is associated with participants’ future survey about their stress. Higher levels of stress is associated with a greater percentage of negative sentiments and percentage of tweets (Liu et al., 2019.) |
| Suicide risk | Problematic Internet use and Internet addiction increase suicide risk in areas of loneliness and negative self-evaluation, hostility (Alpaslan et al., 2015; Kurt, 2015; Poorolajal et al., 2019). |
| ***Physical health and well-being*** |  |
| ***Health-promoting Internet use*** |  |
| Physical activity | The Pokemon GO players who are used to being sedentary, benefit the most from playing. Meaning, the players who never or rarely stay outdoors or walked/jogged before would significantly stay outdoors more frequently as well as walking/jogging more often in order to play Pokémon Go (Wong et al., 2017). |
| Relaxation and leisure | Social media use at least 6 days a week is positively related to relaxation and leisure (Austin-McCain, 2017). |
| Sleep | More diversity in digital media activities is associated with increased total sleep time. Especially using multiple media in the 2 h before bedtime has a protective effect on sleep (Orzech et al., 2016).  WeChat users have better sleep quality in subjective quality of sleep, sleep latency and use of sleeping medication (Xu et al., 2016). |
| ***Health-threatening Internet use*** |  |
| Bedtime | Digital media quantity predicts later bedtime (Orzech et al., 2016). |
| Daytime sleepiness | Students who spend more time on social networking sites by staying up late at night experience greater daytime sleepiness (Nasirudeem et al., 2017). |
| Drug use | Following a reality TV character on Twitter (incl. Internet) have significant increased odds for illegal drug use (Fogel and Shlivko, 2016). |
| Eating and drinking | Students reported eating something and drinking cans of carbonated drinks while using the Internet (Qader et al., 2015) |
| Eating disorders | Social media use reduced physical activity and is a factor in developing eating disorders (Rahman et al., 2020) |
| Eyes | Students with Internet addiction have problems with eyes (Thakur et al., 2017; Rahman et al., 2020). |
| Headaches | As result of social media use headaches were experienced (Rahman et al., 2020) |
| Health status | Students who overuse cell phone have lower general health status (Kawyannejad et al., 2019). |
| Musculoskeletal problems | Students with Internet addiction have more musculoskeletal problems i.e. pain around the region of neck, shoulders, waist, finger and wrist (Thakur et al., 2017). |
| Physical quality of life | Students with Internet addiction have significantly lower health-related quality of life in physical domain (Chern and Huang, 2018). |
| Poor posture | As result of social media use, poor posture was experienced (Rahman et al., 2020) |
| Rest | Students use social networking applications in the last hours of night or after midnight instead of having rest (Mohammadbeigi et al., 2016b). |
| Sedentary behavior | Students are sedentary when using smartphones (Kalirathinam et al., 2017). |
| Self-rated health | Internet addiction is associated with bad self-rated health (Mohammadbeigi et al., 2016a). |
| Sleep | When students’ nighttime Internet usage (texting, social media, gaming, etc.) increases, sleep quality tends to decrease (Whipps et al., 2018).  The longer the students stay up late at night to spend time on social networking sites, the lesser is their hours of sleep (Nasirudeem et al., 2017). Digital media quantity predicts reduced total sleep time (Orzech et al., 2016).Students with Internet addiction have more sleep disturbance (Thakur et al., 2017). |
| Smoking and alcohol use | Students who are smokers and have Internet addiction, have also problematic mobile phone use (incl. Internet). Also, students with problematic mobile phone use are more likely to use alcohol (Tao et al., 2017). |
| Upper extremity and neck symptoms | A high prevalence of upper extremity (shoulder, elbow and lower arm, and hand) and neck symptoms in various positions are noted when using the smartphone (Kalirathinam et al., 2017). |
| Upper respiratory infections | Virtual social network size is significantly related to the rate of upper respiratory infections (Campisi et al., 2017). |
| ***Social health and well-being*** |  |
| ***Health-promoting Internet use*** |  |
| Emotional connections | Students feel emotionally connected when they connect friends on social networking sites (Mahapatra and Schatz, 2015). |
| Engagement with peers | Social media can be used for engagement with peers and for expansion of social networks (Lattie et al., 2019) |
| Offline relationships | The reliance for online interactions does not discourage or limit students offline relationship experience (Mahapatra and Schatz, 2015). |
| Social participation | Social media use at least 6 days a week is positively related to social participation activities (Austin-McCain, 2017). |
| Social support | Social media can be used for social support networks (Lattie et al., 2019) |
| Sense of satisfaction | Students feel an overall sense of satisfaction when they connect friends on social networking sites (Mahapatra and Schatz, 2015). |
| ***Health-threatening Internet use*** |  |
| Face-to-face interactions | Decrease in face-to-face interactions is a proposed negative effect of personal computing technology use (Lattie et al., 2019). |
| Friends | Fewer number of close friends is a significant predictor of excessive Facebook use (Lee et al., 2019). |
| Hyper-connectivity | Hyper-connectivity with peers is a proposed negative effect of personal computing technology use (Lattie et al., 2019). |
| Lower social quality of life | Students with Internet addiction have significantly lower health-related quality of life in social domain (Chern and Huang, 2018). |
| Peer comparison | Peer comparison is a proposed negative effect of personal computing technology use (Lattie et al., 2019). |
| Social skill | Impairment of social skill development is a proposed negative effect of personal computing technology use (Lattie et al., 2019). |
| ***Intellectual health and well-being*** |  |
| ***Health-promoting Internet use*** |  |
| Electronic health literacy | Electronic health literacy is significantly correlated with an individual’s general health, exercise regime, sleep, getting vaccines, and maintenance of sexual health, a balanced diet, stable friendships, and a lifestyle free of harmful substances (Britt et al., 2017). |
| Government or health organization websites | Government or health organizations websites are used as sources when seeking general health information (Schwartz and Richard, 2015). |
| Health information | Internet is used to seek health information. The online health information can be used for decision making and for actions concerning health, such to lifestyle change. The search for health information improves students’ health conditions (Asibey et al., 2017). |
| Health problem | Internet is used to understand a health problem by seeking health information online (Bati et al., 2018). |
| Health professionals | Internet is used for health purposes as for interacting with health professionals (Asibey et al., 2017). Technology-enabled mental health services offer the possibility to expand treatment options and reduce barriers to mental health services (Lattie et al., 2019). |
